# A proximity-labeling map of PI5P4K phosphoinositide kinases interaction networks

**DOI:** 10.1016/j.jbc.2026.113071

**Published:** 2026-04-25

**Authors:** Alicia Llorente, Gurpreet K. Arora, Ryan M. Loughran, Rabi Murad, Svetlana Maurya, Sophia Crabtree, Benji Portillo, Pratima Chapagain, Hundaol Huluka, Scott Eron, Krista Goodman, Guosen Ye, Raymond D. Blind, Brooke M. Emerling

**Affiliations:** 1 Cancer Metabolism and Microenvironment Program, NCI-Designated Cancer Center, Sanford Burnham Prebys Medical Discovery Institute, La Jolla, USA; 2 Bioinformatics Core, Sanford Burnham Prebys Medical Discovery Institute, La Jolla, USA; 3 Proteomics Core, Sanford Burnham Prebys Medical Discovery Institute, La Jolla, USA; 4 Flow Cytometry Core, Sanford Burnham Prebys Medical Discovery Institute, La Jolla, USA; 5 Department of Medicine, Division of Diabetes, Endocrinology and Metabolism, Vanderbilt University Medical Center, Nashville, USA; 6 Larkspur Biosciences, Cambridge, USA

## Abstract

Phosphatidylinositol-5-phosphate 4-kinases (PI5P4Ks) have emerged as candidate drug targets in cancer, neurological, inflammatory, and infectious diseases. Although their canonical function is to phosphorylate PI(5)P to generate PI(4,5)P_2_, growing evidence points to additional catalytic-independent roles, but how these functions are organized within protein interaction networks remains unclear. Here, we use proximity-dependent biotin identification (BioID) in HeLa cells to map isoform-resolved interactomes of human PI5P4Kα, PI5P4Kβ, and PI5P4Kγ. This approach captures PI5P4K-proximal proteins in intact cells and reveals interaction networks positioning these kinases within trafficking-associated signaling modules. Importantly, BioID analysis indicates that PI5P4Kγ has the most extensive set of proximal interactors among the three PI5P4Ks, consistent with its comparatively low catalytic activity and a prominent scaffold-like function. Among PI5P4Kγ-enriched partners, we highlight the endosomal cargo adaptor SNX17 as a proximal interactor that links PI5P4Kγ to β1-integrin recycling and to cell migration and invasion. By providing a proximity map for all three PI5P4Ks, this study offers a framework to help define contexts in which targeted protein degradation may offer advantages over catalytic inhibition and provides a resource for future mechanistic studies on these phosphoinositide kinases.

Phosphatidylinositol-5-phosphate 4-kinases (PI5P4Ks) regulate the interconversion of the phosphoinositides phosphatidylinositol 5-phosphate (PI(5)P) and phosphatidylinositol 4,5-bisphosphate (PI(4,5)P_2_) (1), modulating cellular signaling, metabolism, trafficking events, and stress responses (2). Recent studies have implicated PI5P4Ks in several pathological processes, including neurodegenerative, immunological, and infectious diseases, as well as multiple cancer types (2, 3). Given their translational potential and druggability, there is growing interest in selectively targeting these kinases (4, 5).

The mammalian PI5P4K family comprises three members (PI5P4Kα, PI5P4Kβ, and PI5P4Kγ, encoded by the genes *PIP4K2A*, *PIP4K2B*, and *PIP4K2C*, respectively) with different catalytic activities (3). PI5P4Kα and PI5P4Kβ function as catalytically active lipid kinases, while PI5P4Kγ displays little to no kinase activity *in vitro* and is increasingly thought to act primarily through scaffolding mechanisms (6, 7). PI5P4Ks are predominantly associated with intracellular membranes (8, 9), with the PI5P4Kβ isoform also displaying a well-established nuclear pool (7, 10). Besides phosphoinositide phosphorylation, emerging evidence suggests that catalytic-independent functions contribute to PI5P4K biology (11, 12, 13, 14, 15). This indicates that protein-protein interactions may help organize signaling modules beyond lipid phosphorylation. Understanding these non-catalytic roles is important both for mechanistic biology and for fine-tuning drug-discovery strategies, as certain pathways may remain active when catalytic activity is pharmacologically inhibited.

From a therapeutic perspective, classical small-molecule inhibition of PI5P4Ks has proven challenging. Some inhibitors have been developed (16, 17, 18, 19, 20, 21, 22, 23, 24, 25), but none have advanced to clinical trials. Considering these issues and evidence for non-enzymatic roles, there is growing interest towards targeted protein degradation as an alternative modality. Indeed, PI5P4Kγ degraders (JWZ-1-80 (26), TMX-4153 (27), LRK-4189 [https://acs.digitellinc.com/p/s/discovery-of-lrk-4189-a-first-in-class-degrader-of-the-lipid-kinase-pip4k2c-for-the-treatment-of-microsatellite-stable-mss-colorectal-carcinoma-640250]) now demonstrate that PI5P4Kγ can be degraded through induced proximity in cells, offering a route to eliminate both catalytic and scaffolding functions in one pharmacological move.

Therefore, a comprehensive view of PI5P4K interaction networks in living cells can shed some light on the mediators of their non-catalytic functions. For example, it can reveal interactions with trafficking adaptors and signaling scaffolds that are not necessarily disrupted by active-site inhibition, and it can provide relevant information for choosing between inhibitor-based *versus* degrader-based strategies. Unlike traditional co-immunoprecipitation, proximity-dependent biotin identification (BioID) can capture weak and transient associations in intact cells (28), revealing isoform-specific scaffolds and providing a map of proximity partners.

Here, we use a BioID proximity-labeling strategy to define the proximal interactomes of the three human PI5P4K isoforms. We identify high-confidence shared and isoform-specific partners and uncover functional modules consistent with roles in vesicular trafficking and membrane organization. Given PI5P4Kγ′s weak catalytic activity, its functions are more likely to be mediated through catalytic-independent protein interactions, making its proximal interactome particularly informative. Notably, we discover the endosomal membrane protein SNX17 as a PI5P4Kγ-proximal partner, a previously unreported connection that has important implications for how this kinase regulates cell migration and invasion. We demonstrate that PI5P4Kγ regulates β1-integrin surface levels, integrin-dependent signaling, and cell motility and invasion, consistent with a role in integrin recycling.

Our findings reveal previously unappreciated non-catalytic functions of PI5P4Ks that may support disease-relevant phenotypes and provide a valuable resource of candidate interactors for future mechanistic studies on this kinase family.

## Results

### Identification of high-confidence interactors for PI5P4Ks

**Figure 1:**
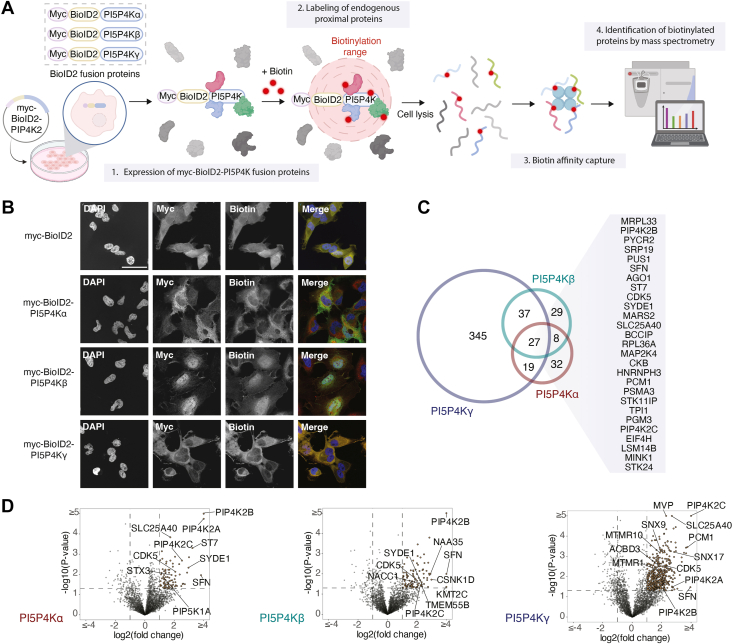
**Proximity-dependent biotin identification (BioID) mapping of the PI5P4Ks interactome.***A*, schematic representation of the BioID experimental workflow. HeLa cells stably expressing BioID2-tagged PI5P4K isoforms (PI5P4Kα, PI5P4Kβ, and PI5P4Kγ) or BioID2 alone were incubated with biotin for 8 h. Biotinylated proteins were captured by streptavidin bead pulldown, digested on-bead, and identified by LC-MS/MS. *B*, representative immunofluorescence images of HeLa cells stably expressing myc-BioID2 or myc-BioID2-PI5P4K proteins. Anti-myc and fluorescently labeled streptavidin were used to visualize bait expression and proximal biotinylation. Colocalization of streptavidin signal with the tagged constructs confirms proximity labeling. Scale bar, 50 μm. *C*, Venn diagrams showing high-confidence PI5P4Ks interactors (≥2-fold enrichment in BioID2–PI5P4K samples relative to myc–BioID2 control and *p* < 0.05) across six independent biological replicates. Shared interactors for PI5P4Kα, PI5P4Kβ, and PI5P4Kγ are highlighted. *D*, Volcano plots showing biotinylated protein abundance for myc-BioID2-PI5P4K proteins relative to myc–BioID2 control. High-confidence interactors above both thresholds are highlighted and selected candidates are labeled.

The BioID technique uses a promiscuous biotin ligase that labels proteins within ∼10 nm of the bait, enabling the identification of interactors in their native cellular environment and capturing weak or transient associations that are difficult to detect with traditional pull-down approaches (28, 29). To capture PI5P4K protein–protein associations, PI5P4K isoforms were fused in-frame to the C terminus of the second-generation biotin ligase BioID2 (29) (myc–BioID2-PI5P4K) and stably expressed in HeLa cells (Fig. 1*A*). Immunoblot analysis confirmed expression of the myc-BioID2-PI5P4K fusion proteins and their ability to biotinylate proximal proteins (Fig. S1*A*). Moreover, their subcellular location was consistent with the reported intracellular membrane association of PI5P4Ks, including the well-established nuclear localization of PI5P4Kβ (Fig. 1*B*). In contrast, the myc-BioID2 biotin ligase alone was broadly distributed throughout the cytoplasm and nucleus.

Streptavidin pull-down from biotin-treated cells expressing myc-BioID2-PI5P4K proteins or the myc-BioID2 control was followed by LC–MS/MS identification of bound proteins. This analysis revealed 86 high-confidence interactors for PI5P4Kα, 101 for PI5P4Kβ, and 428 for PI5P4Kγ, with 27 interactors common to all three family members (Fig. 1, *C* and *D*, and Tables S1-S3). Of note, PI5P4Kγ displayed the largest interactome, which is consistent with its minimal catalytic activity and supports a predominant scaffolding role for this isoform.

Our BioID analysis revealed a network of protein–protein associations containing multiple phosphoinositide-modifying enzymes, including PI4P5Kα (encoded by the *PIP5K1A* gene), a type I phosphatidylinositol 4-phosphate 5-kinase, and phosphoinositide phosphatases such as TMEM55B and members of the myotubularin-related (MTMR) protein family, which regulate phosphoinositide turnover (Fig. 1*D* and Fig. S1*B*). This supports the idea that PI5P4Ks operate within a coordinated lipid-signaling module that regulates phosphoinositide pools. Notably, the interactome also included proteins previously linked to other phosphoinositide kinases, including CDK5 and SFN, as well as trafficking regulators such as SNX9 and ACBD3 (Fig. 1*D*), suggesting that PI5P4Ks may participate in broader membrane-signaling networks. Consistent with this, several interactors across the three isoforms, including SNX17, STX3, and MTMR family members, support enrichment of trafficking- and endomembrane-related functions. In addition, selected PI5P4Kβ-associated proteins, including the nuclear factors KMT2C and NACC1, are compatible with the known nuclear localization of this isoform. In line with previous reports indicating that PI5P4K isoforms can form homo- and heterodimers (30), our BioID dataset recapitulated these reciprocal associations, supporting the validity of the approach. Furthermore, we corroborated these interactions by co-immunoprecipitation (Fig. S1*C*).

### Protein interactome analysis

**Figure 2:**
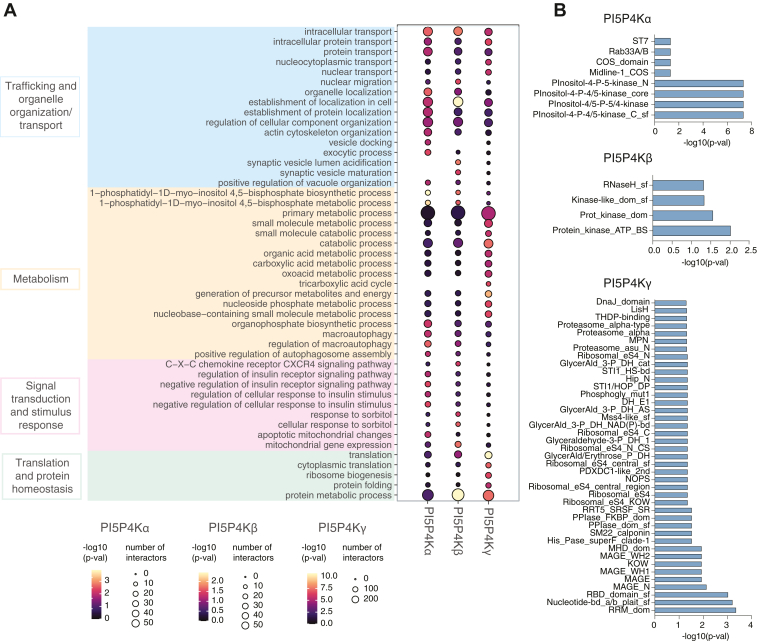
**Proximity-labeling proteomics analysis.***A*, dot plot showing Gene Ontology Biological Process (GO:BP) enrichment for the three PI5P4K baits (PI5P4Kα, PI5P4Kβ, and PI5P4Kγ). The top 20 GO terms for each bait are grouped into functional clusters (trafficking and organelle organization/transport, metabolism, signal transduction and stimulus response, and translation and protein homeostasis). Dot size indicates the number of proteins annotated to each term, and dot color shows −log10(p-val). *B*, bar plots showing enriched InterPro protein domains among significant BioID interactors for each kinase bait (PI5P4Kα, PI5P4Kβ, and PI5P4Kγ). BioID proximity-labeling proteomics was performed in six independent biological replicates.

To obtain a view of the pathways represented within the PI5P4K interactome, we performed Gene Ontology Biological Process (GO:BP) enrichment analysis on high-confidence BioID hits for each isoform (Fig. 2*A*). The top terms for PI5P4Kα, PI5P4Kβ, and PI5P4Kγ clustered into four major functional groups: Trafficking and organelle organization/transport, Metabolism, Signal transduction and stimulus response, and Translation and protein homeostasis. Trafficking and organelle organization related terms were prominently enriched across all baits, consistent with a shared role for PI5P4Ks in regulating endomembrane dynamics. In parallel, enrichment of signaling and metabolism-associated categories is consistent with prior links between PI5P4Ks, stress-response pathways and metabolic regulation (9, 10, 15, 17, 31, 32, 33, 34).

We additionally analyzed Gene Ontology Molecular Function (GO:MF) and Gene Ontology Cellular Component (GO:CC) enrichment to provide functional and subcellular context (Fig. S2, *A* and *B*). GO:MF terms included kinase activity, but also extensive protein binding categories, consistent with non-catalytic protein interactions within these networks. Moreover, GO:CC enriched terms included broad cytoplasmic/cytosolic annotations together with enrichment for endomembrane and vesicle-associated compartments, with a particularly strong signature in PI5P4Kγ. These observations indicate that a large fraction of PI5P4K-proximal proteins map to endosomal and related membrane trafficking organelles and support the idea that PI5P4Ks are part of membrane-trafficking modules.

We next examined the domain architecture of PI5P4K-proximal proteins by performing InterPro domain enrichment analysis on the BioID interactors for each bait (Fig. 2*B*). Enriched domains across PI5P4Kα interactors included catalytic modules from phosphoinositide kinases, consistent with the proximity of other PI4P5K and PI5P4K family members, and scaffold-like domains such as COS/Midline-1 and ST7. For PI5P4Kβ, there was a strong enrichment of protein kinase domains, and the PI5P4Kγ interactome showed an over-representation of RNA-binding and ribosomal modules together with protein–protein interaction scaffolds, including MAGE and LisH domains and cytoskeletal adaptors such as SM22/calponin, as well as co-chaperone and proteasome-associated modules. In contrast, there was no significant enrichment of canonical lipid-binding domains such as PH, PX, C2, ENTH or FYVE across any of the three PI5P4K baits. If proximity labeling were driven mainly by membrane co-residence or indirect lipid-mediated proximity, canonical lipid-binding domains might be expected to be enriched. Instead, their absence suggests that the labeled proteins more likely reflect PI5P4K-centered protein networks rather than broad labeling of lipid-binding proteins. This pattern supports the interpretation that PI5P4Ks, particularly PI5P4Kγ, are embedded within scaffolding networks that coordinate membrane trafficking and signaling.

### PI5P4Kγ interacts with SNX17

**Figure 3:**
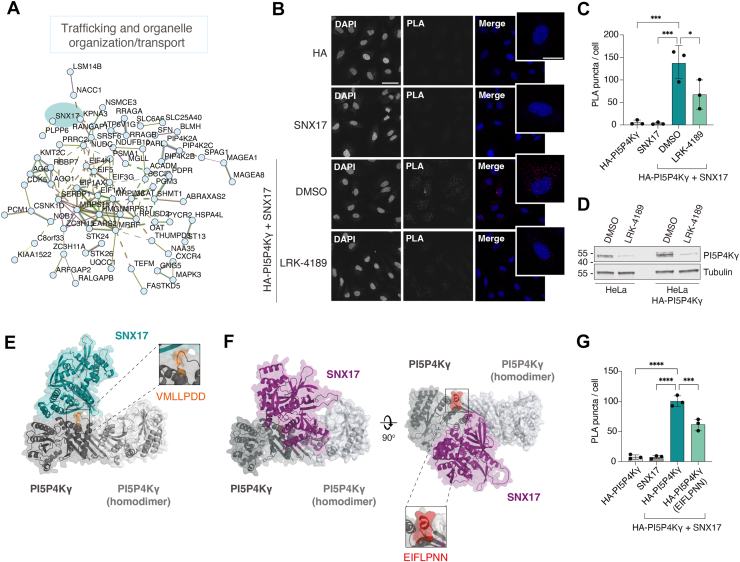
**PI5P4Kγ interacts with the endosomal adaptor SNX17 in HeLa cells.***A*, STRING Network of selected high-confidence PI5P4K interactors involved in trafficking and organelle organization/transport (≥2-fold enrichment in BioID2–PI5P4K samples relative to myc–BioID2 control and *p* < 0.05). Network edges indicate known and predicted protein associations from STRING, including functional relationships derived from available evidence sources. SNX17 is highlighted in the network. Disconnected nodes are hidden. *B*, proximity ligation assay (PLA) showing the proximal association between HA-PI5P4Kγ and SNX17 in HA-PI5P4Kγ overexpressing HeLa cells treated with DMSO or the PI5P4Kγ degrader (±)-LRK-4189 (1 μM, 24 h). Representative confocal images from one of three independent biological replicates showing PLA red puncta and DAPI nuclear staining (with zoomed insets), as well as single antibody controls are shown. Scale bar, 50 μm; inset, 20 μm. *C*, PLA signal quantification. Each dot represents the average PLA puncta per nucleus for a field of view. Bars represent mean ± SD. Statistical significance determined by one-way ANOVA followed by Tukey’s multiple comparisons test (n = 3 biological replicates). *D*, representative immunoblot showing PI5P4Kγ loss after (±)-LRK-4189 treatment (1 μM, 24 h) in HeLa cells and in HA-PI5P4Kγ overexpressing HeLa cells. Tubulin was used as loading control. *E*, top-ranked AlphaFold-Multimer prediction of a putative interface between PI5P4Kγ and SNX17. SNX17 is represented in *green*, and a PI5P4Kγ homodimer in *gray*. The VMLLPDD motif (*orange*) is positioned at the predicted protein–protein interface, and the model predicts that SNX17 does not disrupt PI5P4Kγ dimerization. Inset shows a zoomed-in view of the VMLLPDD motif. *F*, top-ranked AlphaFold-Multimer prediction of a putative interface between mutant PI5P4Kγ (VMLLPDD → EIFLPNN) and SNX17. SNX17 is shown in *purple* and a PI5P4Kγ homodimer in *gray*. The EIFLPNN motif (*red*) is not located at the predicted protein–protein interface in the mutant model. The inset shows a zoomed-in view of the EIFLPNN motif. *G*, quantification of the PLA signal between SNX17 and HA-tagged wild-type or mutant PI5P4Kγ (VMLLPDD→EIFLPNN) in HeLa cells. Each dot represents the average PLA puncta per nucleus for one field of view. Bars represent mean ± SD. Statistical significance was determined by one-way ANOVA followed by Tukey’s multiple comparisons test (n = 3 biological replicates). ∗*p* < 0.05, ∗∗∗*p* < 0.001, and ∗∗∗∗*p* < 0.0001.

Focusing on the trafficking cluster, a STRING network built from selected high-confidence interactors revealed a densely connected set of proteins involved in vesicle transport and organelle organization, with few isolated nodes (Fig. 3*A*), supporting the idea that PI5P4Ks sit within pre-existing trafficking hubs. Based on the BioID2 dataset and GO analyses, we focused on the interaction between PI5P4Kγ and the endosomal adaptor SNX17 for in-depth validation. Among the three PI5P4K isoforms, PI5P4Kγ displayed the broadest interactome, consistent with a less canonical, scaffold-like role rather than a purely catalytic function. At the same time, SNX17 emerged as one of the top high-confidence PI5P4Kγ interactors within the trafficking/endomembrane organization cluster and is a well-established regulator of endosomal recycling of multiple transmembrane cargoes. Together, these features made the PI5P4Kγ–SNX17 pair an attractive candidate to test the idea that PI5P4Kγ organizes protein complexes at endosomal membranes, and we therefore prioritized this kinase–adaptor interaction for experimental validation.

To validate the interaction between PI5P4Kγ and SNX17 in cells, we performed proximity ligation assays (PLA) in HeLa cells overexpressing HA-tagged PI5P4Kγ and treated them with either DMSO or the PI5P4Kγ degrader (±)-LRK-4189 (Fig. 3, *B* and *C*). In DMSO-treated cells, we observed robust PLA puncta, consistent with a proximal association between HA-PI5P4Kγ and endogenous SNX17, whereas single-antibody controls showed minimal background signal. Treatment with (±)-LRK-4189 markedly reduced the PLA signal, suggesting that the interaction depends on PI5P4Kγ protein abundance. Immunoblot analysis verified efficient (±)-LRK-4189–mediated depletion of PI5P4Kγ in both parental and HA-PI5P4Kγ–overexpressing HeLa cells under the same treatment conditions (Fig. 3*D*). Additional PLA assays corroborated BioID-identified interactions between PI5P4K isoforms and other partners, such as SFN (14-3-3σ), a regulatory scaffold protein, and RRAGB, a Rag GTPase linked to nutrient-sensing and mTOR signaling (Fig. S3, *A* and *B*).

To gain structural insight into how PI5P4Kγ might engage SNX17, we used AlphaFold-Multimer to model a putative PI5P4Kγ–SNX17 complex. The resulting prediction revealed a defined interface between SNX17 and PI5P4Kγ, supporting the idea that PI5P4Kγ can form a direct or tightly associated complex with SNX17 (Fig. 3*E*). Interestingly, a PI5P4Kγ loop containing a VMLLPDD sequence is found at the heart of the predicted contact surface. Indeed, four of the five complex models from the AlphaFold-Multimer prediction software had this motif at the heart of the protein-protein interface (Fig. S4). Prior work has shown that PI5P4Kγ binds PI4P5Ks through this VMLLPDD motif, suppressing PI4P5K lipid kinase activity (15). These observations suggest that PI5P4Kγ leverages the same conserved interaction loop implicated in PI4P5K regulation to engage trafficking-associated proteins such as SNX17.

To probe the role of the VMLLPDD motif in SNX17 recognition, we repeated AlphaFold-Multimer modeling using a PI5P4Kγ mutant in which this motif was replaced with EIFLPNN, based on prior work showing that this substitution disrupts the PI5P4K interaction surface required for PI4P5K binding and inhibition (15). Whereas wild-type PI5P4Kγ consistently supported a docking arrangement centered on the VMLLPDD-containing loop (Fig. 3*E* and Fig. S4), the mutant substantially disrupted this predicted interface (Fig. 3*F* and Fig. S5). Monomeric PI5P4Kγ (VMLLPDD→EIFLPNN) failed to yield reasonable complexes with SNX17, and modeling with dimeric PI5P4Kγ (VMLLPDD→EIFLPNN) produced only five low-confidence candidate complexes (Fig. S5). In four of the five models, SNX17 was displaced away from the mutated loop and instead adopted an alternative binding mode similar to the minor outlier configuration observed in the wild-type prediction. The only model retaining SNX17 near the mutant EIFLPNN sequence showed severe steric clashes and was therefore considered structurally implausible (Fig. S6). Together, these observations indicate that the VMLLPDD motif is a key determinant of the preferred PI5P4Kγ-SNX17 interaction surface. To functionally test this prediction, we generated a HA-tagged PI5P4Kγ mutant in which the VMLLPDD motif was replaced with EIFLPNN and repeated the PLA assay with SNX17 (Fig. 3*G* and Fig. S7). Consistent with the modeling results, expression of mutant PI5P4Kγ significantly reduced the PLA signal relative to wild-type PI5P4Kγ, indicating that the VMLLPDD motif contributes to the PI5P4Kγ-SNX17 interaction.

### PI5P4Kγ controls integrin signaling

**Figure 4:**
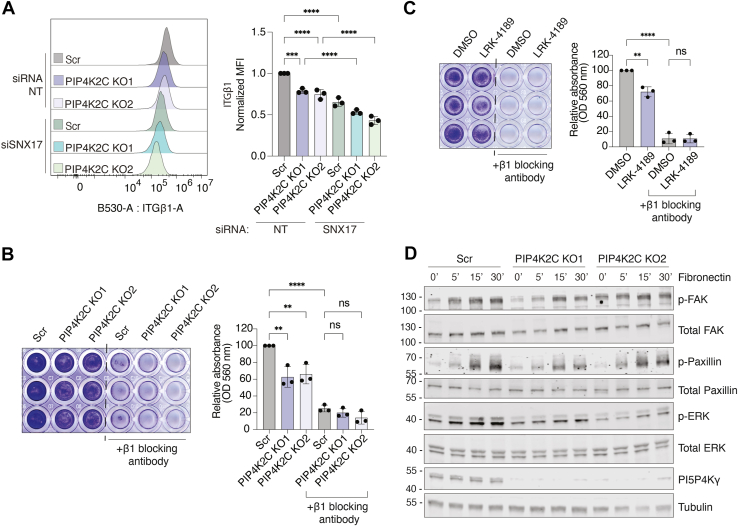
**PI5P4Kγ regulates integrin β1 levels and fibronectin adhesion in HeLa cells.***A*, flow cytometric analysis of surface integrin β1 in scrambled control (Scr) and PI5P4Kγ-depleted HeLa cells following transfection with either non-targeting siRNA (siRNA NT) or siSNX17. The histograms (*left*) show a representative replicate from one of three independent experiments. The bar graph (*right*) shows the median fluorescence intensity (MFI) of surface integrin β1, normalized within each experiment to the Scr siRNA NT control, and plotted as mean ± SD (n = 3 biological replicates). Statistical significance was determined by one-way ANOVA followed by Tukey’s multiple comparisons test. *B*, fibronectin adhesion assay in scrambled control (Scr) and PI5P4Kγ-depleted HeLa cells. Adherent cells were quantified and expressed as percent of Scr control, set to 100% (*right*). An integrin β1–blocking antibody was used as a positive control for impaired adhesion. Statistical significance was determined by one-way ANOVA followed by Tukey’s multiple comparisons test. *C*, fibronectin adhesion assay in HeLa cells treated with DMSO or the PI5P4Kγ degrader (±)-LRK-4189 (1 μM, 24 h). Adherent cells were quantified and expressed as percent of DMSO control, set to 100% (*right*). An integrin β1–blocking antibody was used as a positive control for impaired adhesion. Statistical significance was determined by Student’s *t* test. *D*, representative immunoblots assessing integrin signaling after fibronectin stimulation in Scr and PI5P4Kγ-depleted HeLa cells at the indicated time points. Tubulin was used as a loading control. Western blots are representative of three independent experiments. ns = non-significant, ∗∗*p* < 0.01, ∗∗∗*p* < 0.001, ∗∗∗∗*p* < 0.0001.

Prior work has shown that SNX17 controls endosomal recycling of integrin β1 to the plasma membrane (35, 36). Given this established role and our evidence that PI5P4Kγ forms a complex with SNX17, we next asked whether PI5P4Kγ also modulates integrin surface levels and downstream signaling. To determine whether PI5P4Kγ influences integrin β1 at the cell surface, we first quantified integrin β1 levels by flow cytometry in scrambled control (Scr) and PI5P4Kγ-depleted HeLa cells transfected with either non-targeting siRNA or siSNX17 (Fig. 4*A* and Fig. S8*A*). As expected from the known role of SNX17 in integrin recycling, SNX17 downregulation alone significantly reduced surface integrin β1 levels, and loss of PI5P4Kγ produced a similar effect. Importantly, the reduction was more pronounced when SNX17 was downregulated in both PI5P4Kγ knockout clones, supporting functional cooperation between PI5P4Kγ and SNX17 in the integrin trafficking pathway. Loss of PI5P4Kγ decreased surface integrin β1 without affecting total integrin β1 levels or *ITGB1* mRNA levels (Fig. S8, *B* and *C*), consistent with a defect in integrin trafficking rather than expression. We next asked whether PI5P4Kγ impacts integrin-dependent adhesion to fibronectin. In fibronectin adhesion assays, PI5P4Kγ knockout HeLa cells displayed reduced adhesion compared with Scr controls (Fig. 4*B*). Pharmacologic targeting of PI5P4Kγ with the degrader (±)-LRK-4189 (1 μM, 24 h) phenocopied the genetic depletion. (±)-LRK-4189–treated cells adhered less efficiently to fibronectin than DMSO-treated controls (Fig. 4*C*). To directly examine integrin signaling downstream of fibronectin engagement, we monitored FAK, Paxillin, and ERK phosphorylation over time after fibronectin stimulation in Scr and PI5P4Kγ-depleted cells. Fibronectin induced robust activation of the FAK/Paxillin/ERK axis in Scr cells, whereas the magnitude of these phosphorylation events was significantly lessened in PI5P4Kγ-depleted cells (Fig. 4*D*). Together, these data indicate that PI5P4Kγ regulates integrin β1 surface levels, integrin-dependent adhesion to fibronectin, and downstream signaling in HeLa cells.

### PI5P4Kγ positively regulates cell migration and invasion

**Figure 5:**
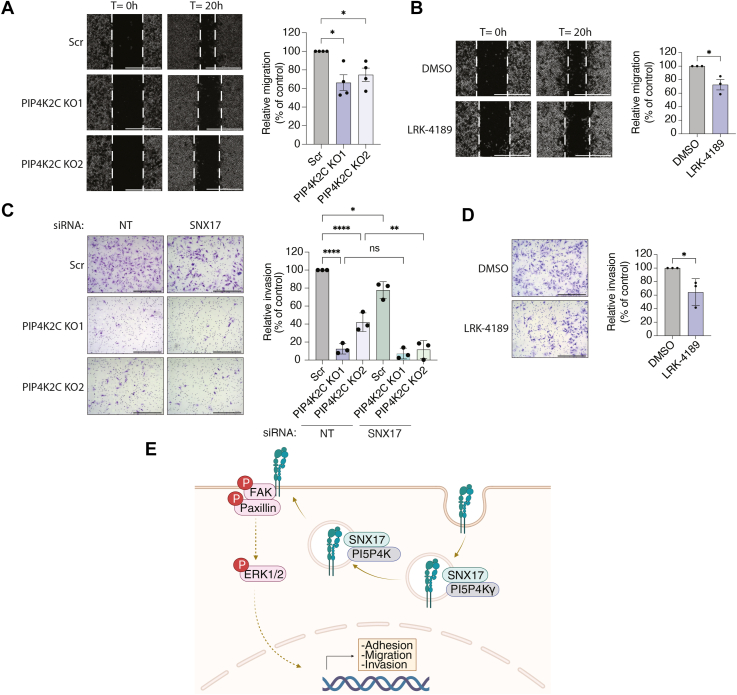
**PI5P4Kγ depletion and degradation impair HeLa cell migration and invasion.***A*, Wound-healing assays performed in confluent monolayers of scrambled control (Scr) and PI5P4Kγ-depleted HeLa cells. Images were acquired immediately after scratching and 20 h later. Wound areas from four independent experiments were quantified and are presented as percent area migrated, with the migrating area of the Scr control set to 100% (*right*). Statistical significance was determined by one-way ANOVA followed by Tukey’s multiple comparisons test. *B*, wound-healing assays performed in confluent monolayers of HeLa cells treated with DMSO or the PI5P4Kγ degrader (±)-LRK-4189 (1 μM, 24 h). Wound areas from three independent experiments were quantified and are presented as percent area migrated, with the migrating area of the DMSO control set to 100% (*right*). Statistical significance was determined by Student’s *t* test. *C*, invasion assays performed in Scr control and PI5P4Kγ-depleted HeLa cells following transfection with either non-targeting siRNA (siRNA NT) or siSNX17. Cells were serum-starved for 6 h and transferred to invasion chambers containing a Matrigel-coated membrane in serum-depleted medium. The bottom chamber contained complete medium to create a chemoattractant gradient. Twenty-four hours after transfer, cells that invaded through the Matrigel were fixed and stained with 0.05% crystal violet. Relative invasion (percent of Scr siRNA NT control set to 100%) is shown (*right*). Statistical significance was determined by one-way ANOVA followed by Tukey’s multiple comparisons test. *D*, invasion assays performed in HeLa cells treated with DMSO or the PI5P4Kγ degrader (±)-LRK-4189 (1 μM, 24 h). Relative invasion (percent of DMSO control set to 100%) is shown (*right*). Statistical significance was determined by Student’s *t* test. Scale bars, 1000 μm. ns = non-significant, ∗*p* < 0.05, ∗∗*p* < 0.01, ∗∗∗∗*p* < 0.0001. *E*, schematic representation of the proposed model for PI5P4Kγ interaction with SNX17. PI5P4Kγ and SNX17 interact at endosomes to regulate cargo recycling, including β1-integrin, back to the plasma membrane. By sustaining β1-integrin surface levels, PI5P4Kγ promotes integrin-dependent signaling and supports cell migration and invasion.

Given the central role of β1-integrin and its associated signaling in cell migration and invasion, we next tested whether PI5P4Kγ loss translates into defects in motility and invasive capacity. To test whether PI5P4Kγ contributes functionally to cell motility, we first examined the impact of PI5P4Kγ depletion on two-dimensional migration using wound-healing assays in confluent HeLa monolayers. Compared with Scr cells, PI5P4Kγ-depleted cells showed delayed wound closure (Fig. 5*A*). Pharmacologic targeting of PI5P4Kγ with the degrader (±)-LRK-4189 similarly impaired wound closure. HeLa cells pre-treated with (±)-LRK-4189 migrated less efficiently than DMSO-treated controls (Fig. 5*B*). We next asked whether PI5P4Kγ also regulates the ability of HeLa cells to invade extracellular matrix. In matrigel-coated invasion chambers, PI5P4Kγ-depleted cells showed reduced invasion toward a serum gradient compared with Scr controls (Fig. 5*C*). SNX17 downregulation alone also impaired invasion, and this phenotype was further enhanced in PI5P4Kγ knockout cells, suggesting that PI5P4Kγ and SNX17 contribute to a shared trafficking mechanism. Although the additional effect of siSNX17 did not reach statistical significance in one PI5P4Kγ knockout clone, this may reflect the already marked reduction in invasion caused by PI5P4Kγ loss alone, which limits the ability to detect a further decrease. Consistently, treatment with (±)-LRK-4189 also led to significantly less invasion relative to DMSO-treated cells (Fig. 5*D*). Together, these data indicate that PI5P4Kγ promotes both migratory and invasive behavior in HeLa cells. In the context of our proximity-labeling data, these findings are consistent with a model in which PI5P4Kγ, in complex with SNX17 at endosomal membranes, supports efficient recycling of cargos, including integrins, back to the plasma membrane to sustain cell migration and invasion (Fig. 5*E*).

## Discussion

This BioID analysis provides isoform-resolved PI5P4K interaction networks and suggests scaffold-like and catalytic-independent roles for these kinases. Each isoform displayed a distinct but partially overlapping interactome, with PI5P4Kγ showing the largest set of high-confidence interactors. Given PI5P4Kγ′s minimal catalytic activity, a broader interactome is consistent with a scaffold-dominant function that organizes signaling and trafficking modules rather than driving lipid turnover alone. Our data further establish PI5P4Kγ as a key regulator of membrane trafficking by showing that loss of PI5P4Kγ impairs integrin-dependent functions such as cell migration and invasion without major changes in *ITGB1* transcript levels, consistent with a defect in integrin trafficking. BioID evidence for PI5P4Kγ and SNX17 proximity, together with altered integrin signaling and associated motility phenotypes in cells lacking PI5P4Kγ, supports a model in which PI5P4Kγ promotes migration and invasion by regulating integrin recycling. In support of this model, combined SNX17 and PIP4K2C downregulation had a synergistic effect on integrin membrane expression and cell invasion, supporting a cooperative role in these processes. Notably, PI5P4Kα and PI5P4Kβ have also been shown to control cell migration and invasion by modulating the Hippo signaling pathway and regulating YAP-dependent pro-migratory gene expression (37). Thus, PI5P4K isoforms converge on the regulation of cell motility through different but complementary mechanisms.

Consistent with reports showing that phosphoinositide kinases from different families can regulate each other, we identified PI4P5Kα as a high-confidence PI5P4Kα interactor in our BioID dataset (Fig. 1*D*, Fig. S2*A*). Recent work has shown that PI5P4Ks can directly associate with PI4P5Ks to restrain PI4P5K dimerization and limit PI(4,5)P_2_ synthesis (15, 38). Our analysis captures the PI5P4Kα-PI4P5Kα proximity, supporting the regulatory interaction between these kinases.

More broadly, BioID revealed several shared partners with phosphoinositide kinases from other families, suggesting the idea of conserved scaffold functions across these enzyme families. For instance, CDK5 emerged as a high-confidence interactor for all three PI5P4Ks in our dataset (Fig. 1, *C* and *D*). Other phosphoinositide kinases have previously been linked to CDK5. In particular, PI4P5Kγ is phosphorylated by CDK5, which reduces its lipid kinase activity and its association with talin at focal adhesions, impacting cell migration and invasion (39, 40). Additionally, SFN (14-3-3σ) was present in our PI5P4K interactome, mirroring observations from other phosphoinositide kinase families in which 14-3-3 proteins play regulatory roles. For example, phosphatidylinositol 4-kinase IIIβ (PI4KIIIβ) directly binds 14-3-3 proteins, which enhances PI4KIIIβ stability and supports its lipid kinase activity (41, 42). Similarly, PI4P5Kγ has been reported to associate with 14-3-3 proteins (43).

Additional PI5P4Kγ interactors common to other phosphoinositide kinases further support a role in the spatial organization of signaling at trafficking compartments. ACBD3 emerged as a PI5P4Kγ interactor, which also plays a role as a scaffold for PI4KIIIβ, recruiting it to Golgi membranes and enhancing its enzymatic activity (41, 44). PI5P4Kγ is associated with MAP4, which was recently shown to directly bind class I PI3Kα, recruiting it to microtubules and promoting its interaction with activated receptors at endosomes to initiate PI3K–AKT signaling (45, 46). PI5P4Kγ was also proximal to the Src-family kinase LYN, which has been shown to associate with class I PI3K, promoting its activation (47).

Moreover, STK11IP was identified as a high-confidence interactor for all three PI5P4K isoforms. STK11IP is named for its interaction with the tumor-suppressor kinase LKB1 (STK11). Interestingly, a recent genome-wide CRISPR screen demonstrated that LKB1 suppresses tumor spheroid growth by activating the lipid kinase PIKfyve and promoting EGFR internalization (48). This suggests that a common mechanism may exist in which LKB1-associated scaffolding proteins coordinate phosphoinositide kinases to regulate membrane trafficking.

Our BioID analysis revealed that PI5P4Kγ associates with multiple sorting nexins, including SNX1, SNX8, SNX9, and SNX17. This aligns with prior reports showing that other phosphoinositide kinases engage SNX-based endosomal trafficking machinery. For example, all PI4P5Ks interact with SNX9 to control membrane remodeling and fission (49), and PI4P5Kγ directly binds SNX5 and SNX6 to regulate EGFR and E-cadherin endosomal sorting (50, 51). Likewise, the class III PI3K VPS34 and PIKfyve cooperate with the SNX17–Retriever–CCC–WASH recycling machinery to regulate integrin recycling (36). Thus, our interactome data place PI5P4Ks within conserved scaffolding networks that couple phosphoinositide metabolism to endosomal trafficking and signaling.

The trafficking role of PI5P4Ks goes beyond the migration and invasion phenotypes described here. Recent work from our group has shown that these kinases also regulate nutrient and cholesterol handling through multiple trafficking pathways. In pancreatic cancer cells, PI5P4Kα supports metabolic fitness by sustaining *GLUT1* expression and surface levels of the transferrin receptor to promote glucose and iron uptake (52). In p53-deficient cells, PI5P4Kα and PI5P4Kβ facilitate lysosomal cholesterol export and proper lysosome positioning to sustain mTOR signaling (53). An earlier study further demonstrated that PI5P4Ks regulate the trafficking of lipid droplets to peroxisomes to sustain mitochondrial homeostasis (34). Although at least some of these trafficking events depend on PI5P4K catalytic activity and its lipid product, our BioID data provide a framework to continue dissecting the multiple functions of this kinase family and to identify additional scaffold-like roles that may contribute to these trafficking processes.

PI5P4Ks have been implicated in diverse human diseases, including multiple cancer types, diabetes, infectious and inflammatory diseases, and neurodegenerative disorders (2, 3, 4). To date, efforts to pharmacologically target PI5P4Ks have mainly resulted in preclinical tool compounds (16, 17, 18, 19, 20, 21, 22, 23, 24, 25). PI5P4K inhibitors have helped validate these kinases as metabolic and stress-response vulnerabilities, but limitations in potency, selectivity and pharmacokinetics have prevented clinical advancement (4). Our interactome maps have important implications for therapeutic targeting because if PI5P4Ks have substantial catalytic-independent functions, then selective protein degradation may be more effective than catalytic inhibition alone. Small-molecule inhibitors may fail to fully suppress PI5P4K-driven signaling pathways, whereas degraders would block both enzymatic and protein-protein interaction functions. In line with this idea, potent PI5P4Kγ degraders have recently been developed (26, 27). Of particular therapeutic significance, a first-in-class PI5P4Kγ-targeting PROTAC degrader ((±)-LRK-4189 [https://acs.digitellinc.com/p/s/discovery-of-lrk-4189-a-first-in-class-degrader-of-the-lipid-kinase-pip4k2c-for-the-treatment-of-microsatellite-stable-mss-colorectal-carcinoma-640250]), is in early clinical development.

By defining isoform-specific interaction networks and highlighting PI5P4Kγ as a scaffold for trafficking and signaling hubs, our study provides a mechanistic framework to guide the clinical development of PI5P4K-targeted degraders and offers tools to further dissect the multiple functions of these kinases.

## Methods

### Cell lines

HeLa cells were obtained from the American Type Culture Collection and were cultured in Dulbecco’s Modified Eagle’s Medium (DMEM 10-013-CV, Corning) supplemented with 10% fetal bovine serum (FBS 89510-186, Avantor) and 100 units/mL penicillin/streptomycin (15140122, Life Technologies). Cells were maintained in standard conditions (37 °C, 5% CO_2_), split every 2 to 3 days and routinely tested for *mycoplasma* contamination using the PCR *Mycoplasma* Detection Kit (G239, ABM). Cell line authentication was performed by the Genomics core at Sanford Burnham Prebys using STR analysis.

### Antibodies

The following antibodies were used: PI5P4Kα (12469-1-AP, Proteintech), PI5P4Kβ (9694S, Cell Signaling Technology), PI5P4Kγ (17077-1-AP, Proteintech), Myc-tag (2276S, Cell Signaling Technology), α-tubulin (66031-1-Ig, Proteintech), HA (H3663, Sigma-Aldrich), SNX17 (10275-1-AP, Proteintech), FAK (13009, Cell Signaling Technology), Phospho-FAK (Tyr576/577) (3281, Cell Signaling Technology), Paxillin (2542, Cell Signaling Technology), Phospho-Paxillin (2541, Cell Signaling Technology), ERK1/2 (9102, Cell Signaling Technology), Phospho-ERK1/2 (Thr202/Tyr204) (9101, Cell Signaling Technology), β-Actin (sc-517582, Santa Cruz Biotechnology), Integrin β1 Alexa Fluor 488-conjugated (ab202641, Abcam), Streptavidin Alexa Fluor 546-conjugated (S11225, Invitrogen), VRDye 549 Streptavidin (926-54030, LI-CORbio), IRDye 800CW goat anti-rabbit igG secondary antibody (926-32211, LI-CORbio), IRDYE 680LT goat anti-mouse igg secondary antibody (926-68020, LI-CORbio), Goat anti-Rabbit IgG Secondary Antibody, Alexa Fluor 488 (A32731, Thermo Fisher Scientific). Antibody specificity was validated using manufacturer information, prior literature where available, and appropriate experimental controls for each application.

### Constructs, viral production, and transduction

For HA-PI5P4Ks overexpression, 3xHA-tagged *PIP4K2A*, 3xHA-tagged *PIP4K2B*, HA-tagged *PIP4K2C*, and HA-tagged mutant *PIP4K2C* (VMLLPDD→EIFLPNN) were PCR-amplified using primers containing NheI and EcoRI restriction sites and cloned into the pLJM1 vector (Addgene plasmid #91980, https://www.addgene.org/91980/) using NheI and EcoRI restriction enzymes (R3131S and R3101S, New England Biolabs), Phusion Hot Start II DNA Polymerase (F549S, Thermo Fisher Scientific) and In-Fusion Snap Assembly Master Mix (638947, Takara).

For BioID constructs, PIP4K2 isoforms were PCR-amplified using primers containing XhoI and SalI restriction sites and cloned into the myc-BioID2-pBABE-puro vector (Addgene plasmid #80900, https://www.addgene.org/80900/) using XhoI and SalI restriction enzymes (R0146S and R3138S, New England Biolabs) and the same cloning workflow described above.

For lentiviral production, human embryonic kidney 293T cells (HEK 293T) were seeded at 80% confluence in 10-cm plates and transfected with lentiviral vector DNA and the packaging plasmids psPAX2 (12260, Addgene) and pMD2.G (12259, Addgene) combined at a ratio of 4:3:1, respectively, using Lipofectamine 2000 (11668019, Life Technologies). For retroviral production, the packaging plasmid used was pCL-Ampho (NBP2-29541, Novus Biologicals).

After 24 h, the medium was replaced with fresh 10% FBS DMEM. The viral supernatant was collected after 48 h, passed through a 0.45-μm cellulose acetate filter, and used to transduce HeLa cells in the presence of 8 μg/ml polybrene (NC9840454; Santa Cruz Biotechnology), followed by puromycin (NC9138068, Invivogen) selection.

### CRISPR/Cas9 *PIP4K2C* knockout

*PIP4K2C* knockout HeLa cells were generated using the human *PIP4K2C* sgRNA CRISPR/Cas9 All-in-One Lentivector set (LG136710, Applied Biological Materials). sgRNA target sequences: TGGAGGCGAAGCCGAAACCT, ATCTGGCAGCAGCATCACCG). Lentiviruses were produced in HEK 293T cells, and the viral supernatant was used to transduce HeLa cells as described above. Transduced cells were selected with puromycin to enrich for edited populations. Single-cell clones were isolated by limiting dilution, expanded, and screened for loss of *PIP4K2C* expression by immunoblotting.

### siRNA transfection

HeLa cells were transfected with SNX17-targeting SMARTpool siRNA (Dharmacon) or a non-targeting control siRNA using Lipofectamine RNAiMAX (Invitrogen) according to the manufacturer’s protocol. Immunoblot analysis was performed 48 h after transfection to confirm knockdown efficiency. Target sequences included in the SMARTpool were: CAUGCAAGCUGUUCGGCAA, AUGCGAUGCUGGCGGGUCA, CUUUAUGCUCAGACGGUAU, CCAGUGAUGUCCACGGCAA.

### Immunoblot analysis

Cells were washed with ice-cold phosphate-buffered saline (PBS) and resuspended in lysis buffer (50 mM Tris/HCl pH 7.4, 1% Triton X-100, 150 mM NaCl, 1 mM EDTA, 1 mM EGTA, 0.1% SDS) containing a protease and phosphatase inhibitor cocktail (78440, Thermo Fisher Scientific). Samples were incubated for 30 min on ice and centrifuged at 21,000*g* for 10 min at 4 °C. The supernatant was kept as the protein extract and concentration was determined by a bicinchoninic acid (BCA) assay (23225, Thermo Scientific). Protein lysate (30 μg per sample) was mixed with sample buffer (45 mM Tris pH 6.8, 10% glycerol, 1% SDS, 52 mM dithiothreitol (DTT) and 1% bromophenol blue) and heated at 95 °C for 5 min. Proteins were separated by sodium dodecyl sulfate polyacrylamide gel electrophoresis (SDS–PAGE) and transferred to nitrocellulose membranes. Membranes were blocked in 5% bovine serum albumin (BSA) (97061-422, VWR) in TBS-Tween (0.1%) for 1 h at room temperature and probed overnight at 4 °C with primary antibodies. Membranes were then washed three times with TBS-Tween (0.1%), incubated for 45 min at room temperature with the appropriate secondary antibodies, and visualized and quantified using a LI-COR Odyssey Imager.

For integrin signaling assays, HeLa cells were serum-starved for 4 h and then stimulated with 10 μg/mL fibronectin (354008, Corning) for the indicated times before lysis.

### Immunofluorescence

Cells were seeded on 96-well optical-grade plastic plates. The next day, cells were fixed with 4% methanol-free paraformaldehyde (PI28908, Fisher Scientific) in PBS for 15 min at room temperature. Cells were then permeabilized with 0.5% Triton X-100 in PBS for 10 min and blocked in 0.3% Triton-X with 5% normal goat serum (01-6201, Thermo Fisher Scientific) for 1 h at room temperature. Primary antibodies were diluted 1:100 in antibody dilution buffer (0.3% TritonX-100 with 1% BSA in PBS) and incubated overnight at 4 °C. Cells were washed three times with blocking buffer for 5 min each, and secondary antibodies diluted 1:500 in antibody dilution buffer were added for 1 h at room temperature. Primary antibodies were detected using Alexa Fluor 488-conjugated secondary antibodies and biotinylated proteins were detected using Alexa Fluor 546-conjugated streptavidin. DAPI was added in an additional blocking buffer wash for 10 min to stain the nuclear compartment. Confocal images were taken on a Zeiss LSM710 microscope.

### Proximity-dependent biotin identification (BioID)

Human HeLa cells stably expressing mycBioID2-PI5P4Ks or the myc-BioID2 control were cultured in five 15-cm plates per condition until reaching 70% confluence, followed by treatment with 50 μM biotin (29129, Thermo Fisher Scientific) for 8 h. The experiment was performed twice, each time including three biological replicates.

Trypsinized cell pellets were washed with ice-cold PBS and dissolved in 8 M urea, 50 mM ammonium bicarbonate (ABC) and benzonase, and the solution was centrifuged at 14,000*g* for 15 min to remove cellular debris. Supernatant protein concentration was determined using a BCA protein assay (23225, Thermo Scientific). Disulfide bridges were reduced with 5 mM tris(2-carboxyethyl)phosphine (TCEP) at 30 °C for 60 min, and cysteines were subsequently alkylated with 15 mM iodoacetamide (IAA) in the dark at room temperature for 30 min.

Affinity purification was carried out in a Bravo AssayMap platform (Agilent) using AssayMap streptavidin cartridges (Agilent). Briefly, cartridges were first primed with 50 mM ammonium bicarbonate, and then proteins were slowly loaded onto the streptavidin cartridge. Background contamination was removed with 8 M urea, 50 mM ammonium bicarbonate. Finally, cartridges were washed with Rapid digestion buffer (Promega, Rapid digestion buffer kit) and proteins were subjected to on-cartridge digestion with mass spec grade Trypsin/Lys-C Rapid digestion enzyme (Promega, Madison, WI) at 70 °C for 1 h. Digested peptides were then desalted in the Bravo platform using AssayMap C18 cartridges and dried down in a SpeedVac concentrator.

Prior to LC-MS/MS analysis, dried peptides were reconstituted with 2% ACN, 0.1% FA and concentration was determined using a NanoDropTM spectrophotometer (Thermo Fisher). Samples were then analyzed by LC-MS/MS using a Proxeon EASY-nanoLC system (Thermo Fisher) coupled to an Orbitrap Fusion Lumos Tribrid mass spectrometer (Thermo Fisher Scientific). Peptides were separated using an analytical C18 Aurora column (75 μm × 250 mm, 1.6 μm particles; IonOpticks) at a flow rate of 300 nL/min (60 °C) using a 75-min gradient: 2% to 6% B in 1 min, 6% to 23% B in 45 min, 23% to 34% B in 28 min, and 34% to 48% B in 1 min (A = FA 0.1%; B = 80% ACN: 0.1% FA). The mass spectrometer was operated in positive data-dependent acquisition mode. MS1 spectra were measured in the Orbitrap in a mass-to-charge (m/z) of 375 to 1500 with a resolution of 60,000. Automatic gain control target was set to 4 × 10^5^ with a maximum injection time of 50 ms. The instrument was set to run in top speed mode with 1-s cycles for the survey and the MS/MS scans. After a survey scan, the most abundant precursors (with charge state between +2 and +7) were isolated in the quadrupole with an isolation window of 0.7 m/z and fragmented with HCD at 30% normalized collision energy. Fragmented precursors were detected in the ion trap as rapid scan mode with automatic gain control target set to 1 × 10^4^ and a maximum injection time set at 35 ms. The dynamic exclusion was set to 20 s with a 10 ppm mass tolerance around the precursor.

All mass spectra were analyzed with MaxQuant software version 2.4.10.0. MS/MS spectra were searched against the *Homo sapiens* UniProt protein sequence database (downloaded in March 2022), and GPM cRAP sequences (commonly known protein contaminants). Precursor mass tolerance was set to 20 ppm and 4.5 ppm for the first search where initial mass recalibration was completed and for the main search, respectively. Product ions were searched with a mass tolerance of 0.5 Da. The maximum precursor ion charge state used for searching was seven. Carbamidomethylation of cysteine was searched as a fixed modification, while oxidation of methionine and acetylation of protein N-terminal were searched as variable modifications. Enzyme was set to trypsin in specific mode and a maximum of two missed cleavages was allowed for searching. The target-decoy-based false discovery rate (FDR) filter for spectrum and protein identification was set to 1%. Label-free quantification was performed using MaxQuant intensity values.

Protein identification results were further processed using the Perseus software suite version 2.0.9.0 (54). The raw intensity measurements for the pulldowns were log transformed (base 2) and subsequently divided into three datasets each. All three datasets had the control measurements and one of the PI5P4Kα/PI5P4Kβ/PI5P4Kγ datasets. This was done to ensure better filtering in the next step. All the proteins found in ≥3 replicates out of six in the PI5P4Kα/PI5P4Kβ/PI5P4Kγ were retained and the rest of the proteins were filtered out. In addition, proteins were retained for downstream analysis if they showed sequence coverage ≥5% or were supported by >2 unique peptides. The numbers of unique peptides and MS/MS counts supporting each protein identification are reported in Tables S1-S3. Imputation was carried out only in the control dataset by random sampling from a left-shifted normal distribution according to default parameters in Perseus. The datasets were normalized by subtracting the median values and the volcano plots were made in R. Proteins were considered significantly enriched if they showed a log2 difference (bait *versus* control) > 1 and *p* < 0.05.

### GO enrichment analysis

To perform gene ontology (GO) enrichment analysis (Biological Process, BP; Molecular Function, MF; and Cellular Component, CC), gene lists containing symbols and corresponding fold-change values for PI5P4Kα, PI5P4Kβ, and PI5P4Kγ interactors were prepared and saved as plain text files. In R, the required packages (clusterProfiler, org.Hs.eg.db, enrichplot, and gprofiler2) were installed and loaded, and *Homo sapiens* was specified as the reference organism to ensure accurate gene-ID mapping. GO enrichment for each kinase bait was conducted using the enrichGO function in clusterProfiler, specifying the appropriate ontology (BP, MF, or CC). To visualize shared and distinct functional patterns, the top 15 or 20 enriched GO terms for each kinase (ranked by adjusted *p*-value) were combined and displayed as dotplots. The size of dots represents the number of interactors associated with each GO term and colors represent −log10 of enrichment *p*-values. Dotplots were generated in R version 4.5.1 using ggplot2 version 3.5.2.

### Network visualization

Protein–protein interaction data were downloaded from the STRING v11 database (55) with the following settings: meaning of network edges—confidence; active interaction sources—textmining, experiments, and databases; minimum required interaction score—0.3. Disconnected nodes in the network were hidden.

### Protein domain enrichment analysis

Significant interactors identified for PI5P4Kα, PI5P4Kβ, and PI5P4Kγ were compiled into separate gene lists. Protein domains were annotated using UniProt identifiers mapped to the InterPro database to identify functional domains enriched among high-confidence interactors.

### Co-immunoprecipitation

HeLa cells expressing HA-tagged PI5P4Ks or an empty plasmid control were collected in lysis buffer (50 mM Tris-HCl pH 7.4, 100 mM NaCl, 1% NP-40, 1 mM EDTA, 1 mM EGTA) supplemented with a protease and phosphatase inhibitor cocktail (78440, ThermoFisher Scientific), incubated for 30 min on ice, and centrifuged at 21,000*g* for 10 min at 4 °C. The supernatant was kept as a protein extract, and the concentration was determined using a BCA assay (23225, Thermo Fisher Scientific). The input (20 μg of protein lysate) was mixed with sample buffer (45 mM Tris pH 6.8, 10% glycerol, 1% SDS, 52 mM DTT and 1% bromophenol blue) and heated at 95 °C for 5 min before immunoblot analysis, then 1 mg of the remaining protein lysate was incubated with 25 μl of anti-HA magnetic beads (88836, ThermoFisher Scientific) overnight at 4 °C with gentle agitation. The following day, the beads were washed five times with lysis buffer using a magnetic stand and eluted by boiling with 2× sample buffer. Proteins were separated by SDS–PAGE before immunoblot analysis.

### Proximity ligation assay

Cells were seeded on 96-well optical-grade plastic plates. The following day, cells were fixed with 4% methanol-free paraformaldehyde (PI28908, Fisher Scientific), permeabilized with 0.5% Triton X-100 in PBS, blocked, and probed with primary antibodies overnight at 4 °C. PLA experiments were performed with Duolink PLA reagents (Sigma-Aldrich) following the manufacturer’s instructions. Briefly, after primary antibody incubation, cells were washed and incubated with the appropriate PLUS and MINUS PLA probes for 1 h at 37 °C. Following additional washes, ligation solution was added and samples were incubated for 30 min at 37 °C. Cells were then washed and subjected to the amplification reaction for 100 min at 37 °C. After final washes, nuclei were counterstained with DAPI and confocal images were taken on the Zeiss LSM 980 Airyscan2 microscope.

The number of PLA puncta per nucleus was quantified by counting at least 85 cells per condition across three independent experiments. Puncta detection was performed in Fiji using a modified version of a tailor-made macro previously described in (56). Briefly, the macro segmented the nuclei by thresholding the DAPI signal and using the Analyze Particles ImageJ plugin, and puncta were detected in the PLA channel using the Find Maxima plugin.

### LRK-4189 PROTAC for targeted degradation

The PI5P4Kγ PROTAC degrader LRK-4189 (Larkspur Biosciences) was prepared in DMSO. Cells were treated with either vehicle control (DMSO) or 1 μM LRK-4189 for 24 h prior to immunoblotting, PLA analyses, fibronectin adhesion assays, and migration and invasion assays.

### Flow cytometry

To measure surface levels of β1-integrin, HeLa cells were seeded in 6-well plates in triplicate. After 24 h, cells were serum-starved for 6 h and then stimulated with 10 μg/mL human fibronectin (354008, Corning) diluted in PBS for 30 min at 37 °C. Then, cells were washed once with ice-cold PBS, dissociated, and stained with the viability dye Ghost Dye Red 710 (13–0871-T500, Cytek Biosciences) and Alexa Fluor 488–conjugated integrin β1 antibody (ab202641, Abcam) for 30 min at 4 °C. Following two PBS washes, cells were fixed with 4% paraformaldehyde for 15 min at room temperature, pelleted, and resuspended in PBS. Data were acquired on a NovoCyte flow cytometer (Agilent Technologies) using NovoExpress software, with daily quality control performed according to the manufacturer’s protocol. For each run, compensation for Ghost Dye Red 710 and Alexa Fluor 488 was performed using single-color controls. A total of 10,000 singlet events were collected per sample, and data were analyzed using FlowJo v10.10.0. Dead cells were excluded by gating out Ghost Dye Red 710–positive events. Median fluorescence intensity (MFI) was quantified and normalized to the Scr control.

### Fibronectin adhesion assay

Fibronectin-coated 96-well plates were prepared by incubating wells with 10 μg/ml fibronectin (354008, Corning) diluted in PBS for 1 h at 37 °C, followed by blocking with 1% BSA in PBS for 1 h at room temperature. HeLa cells were serum-deprived for 6 h, trypsinized, washed once with PBS, and 1 × 10^5^ cells were added per well, with triplicates for each condition. Cells were allowed to adhere for 30 min at 37 °C and non-adherent cells were removed by PBS washing. Adherent cells were fixed with 4% paraformaldehyde for 15 min, washed with PBS, and stained with 0.5% crystal violet (C0775, Sigma) for 20 min. After removing excess crystal violet solution, plates were washed thoroughly by submersion in a water bath and air-dried overnight. Bound crystal violet was dissolved in 0.1 M sodium citrate 25% ethanol pH 4.2, and absorbance was measured at 590 nm on a Varioskan LUX Multimode Microplate Reader (Thermo Fisher Scientific).

### Wound-healing migration assay

Fibronectin-coated 24-well plates were prepared by incubating wells with 10 μg/ml fibronectin (354008, Corning) diluted in PBS for 1 h at 37 °C. HeLa cells were seeded onto the coated plates and grown to full confluency, with six technical replicates per condition. After 24 h, scratches were generated using the AutoScratch Wound Making Tool (BioTek). Cells were washed thoroughly with PBS to remove debris, and fresh 10% FBS DMEM was added. Wound closure was monitored for 20 h on a Cytation 5 imager (BioTek) using the Scratch Assay App. Wound area was quantified using Fiji.

### Invasion assays

Invasion assays were performed using BioCoat Matrigel Invasion Chambers (354480, Corning) following the manufacturer’s instructions. Briefly, HeLa cells were serum-deprived for 6 h. Prior to cell seeding, invasion inserts were rehydrated with serum-free media for 2 h in a humidified tissue culture incubator at 37 °C. Cells were resuspended in serum-free media at 1 × 10^5^ cells/ml. Chemoattractant media (DMEM containing 10% FBS) was added to the wells of the companion 24-well plate, and the rehydrated inserts were transferred into place. Cells were added to the upper chamber (inserts) and incubated overnight to allow invasion through the Matrigel matrix.

Following incubation, cells were fixed with 70% ethanol for 20 min at room temperature, washed with water, and stained with 0.05% crystal violet for 30 min. Excess stain was removed by thorough washing with water, and non-invading cells remaining on the upper surface of the membrane were gently removed with a cotton swab. Membranes were allowed to air dry for at least 24 h before being mounted on microscopy slides. Images of invaded cells were acquired using an Olympus microscope.

### Molecular docking of PI5P4Kγ and SNX17 using AlphaFold multimer

AlphaFold2 (57, 58, 59) was used to predict the protein complex model of PI5P4Kγ and SNX17. AlphaFold Multimer was run using MMseqs2 with default parameters. Protein sequences were input as FASTA files from https://www.uniprot.org/ and generated five potential structural models. The top-ranking structure was chosen based on the predicted interface TM-score. For illustration purposes, the loops with low pLDDT scores (<60) were omitted from the protein complex model. All molecular structures and complexes were generated using Pymol (Schrödinger).

### Statistical analysis

Statistical analyses were performed using R and GraphPad Prism 10 (version 10.3.0), as indicated in each figure legend. Data are presented as the mean of at least three independent biological replicates ± standard deviation (SD), unless otherwise specified. Comparisons between 2 groups were analyzed using two-tailed Student’s t-tests, whereas analyses involving more than 2 groups were assessed by one-way ANOVA followed by Tukey’s multiple comparisons test for multiple comparisons. Statistical significance is indicated in the figures. ∗*p* < 0.05, ∗∗*p* < 0.01, ∗∗∗*p* < 0.001, and ∗∗∗∗*p* < 0.0001. Exact *p*-values for all statistical comparisons are provided in Table S4. For GO and protein domain enrichment analysis, a hypergeometric test with Benjamini-Hochberg correction to control the false discovery rate was applied.

## Data availability

The mass spectrometry proteomics data are available *via* ProteomeXchange with identifier PXD077030.

## Supporting information

This article contains supporting information.

## Conflict of interest

The authors declare the following financial interests/personal relationships which may be considered as potential competing interests: Krista Goodman and Guosen Ye are employees of Larkspur Biosciences, Inc. with stock options. Scott Eron and Brooke M. Emerling are consultants for Larkspur Biosciences, Inc.
