# Supplementary material for: A proximity-labeling map of PI5P4K phosphoinositide kinases interaction networks
Source: J Biol Chem. 2026 Apr 25;302(6):113071. doi: 10.1016/j.jbc.2026.113071 (PMC13218151; doi:10.1016/j.jbc.2026.113071)
Supplement: Supplementary Figure legends [file mmc1.docx]

**SUPPLEMENTARY FIGURE LEGENDS**

**Supplementary Figure 1. Validation of BioID fusion proteins expression and identification of phosphoinositide-modifying enzymes in the PI5P4K interactome. A.** Immunoblots showing expression of myc-BioID2 (control) and myc-BioID2-tagged PI5P4K proteins after 8 h biotin supplementation. Global protein biotinylation was detected using fluorescently labeled streptavidin. Tubulin was used as loading control. **B.** Network diagram highlighting phosphoinositide-modifying enzymes identified within the PI5P4K interactome. **C.** Representative immunoblot of anti-HA co-immunoprecipitations from HeLa cells expressing HA-tagged PI5P4Ks, probed for endogenous PI5P4K proteins and HA.

**Supplementary Figure 2. Gene Ontology enrichment analysis of high-confidence PI5P4K interactors. A.** Dot plot showing Gene Ontology Molecular Function (GO:MF) enrichment for the three PI5P4K baits (PI5P4Kα, PI5P4Kβ, and PI5P4Kγ). The top 15 GO terms for each bait are grouped into functional clusters (Molecular binding and Enzymatic activity). **B.** Dot plot showing Gene Ontology Cellular Component (GO:CC) enrichment for the three PI5P4K baits (PI5P4Kα, PI5P4Kβ, and PI5P4Kγ). The top 20 GO terms for each bait are grouped into functional clusters (Cytoplasm/cytosol, Endomembrane system, Mitochondria, Nucleus, Extracellular, and Other). Dot size indicates the number of proteins annotated to each term, and dot color shows -log10(p-val).

**Supplementary Figure 3. Proximity ligation assay validation of high-confidence PI5P4K interactors. A.** Proximity ligation assay (PLA) validating the proximal association between PI5P4Kγ and SFN in HeLa cells overexpressing the myc-BioID2 control or myc-BioID2-PI5P4K proteins. **B.** Proximity ligation assay (PLA) validating the proximal association between PI5P4Kγ and RRAGB in HeLa cells overexpressing the myc-BioID2 control or myc-BioID2-PI5P4Kγ. Representative confocal images of PLA red puncta and DAPI nuclear staining (with zoomed insets), as well as single-antibody controls, are shown. Scale bar, 50 μm; inset, 20 μm.

**Supplementary Figure 4. AlphaFold-Multimer modeling predicts a PI5P4Kγ-SNX17 interface involving the VMLLPDD motif. A-E.** The five top-ranked AlphaFold-Multimer predictions for a putative PI5P4Kγ-SNX17 complex. In four of the five predicted complexes (A, C, D and E), the VMLLPDD motif (highlighted in orange) is positioned at the predicted protein–protein interface, and the model predicts that SNX17 does not disrupt PI5P4Kγ dimerization. **F.** Overlay of the 4 most similar predictions (A, C, D and E).

**Supplementary Figure 5. AlphaFold-Multimer modeling of mutant PI5P4Kγ-SNX17 predicts loss of interface positioning of the EIFLPNN motif. A-E.** The five top-ranked AlphaFold-Multimer predictions for a mutant PI5P4Kγ (VMLLPDD🡪EIFLPNN)–SNX17 complex. Unlike the wild-type model, in four of the five predicted complexes (A, B, D and E), the EIFLPNN motif (highlighted in red) is not located at the predicted protein–protein interface. **F.** Overlay of the 4 most similar predictions (A, B, D and E).

**Supplementary Figure 6. The only AlphaFold-Multimer model retaining SNX17 near the mutant EIFLPNN sequence reveals severe steric clashes at the putative interface.**
The only AlphaFold-Multimer prediction in which SNX17 remains positioned near the mutant PI5P4Kγ EIFLPNN sequence (highlighted in red) is shown. Although SNX17 remains in proximity to the mutant motif in this model, the predicted arrangement shows severe steric clashes at the putative interface, suggesting that this configuration is unlikely to support a stable interaction. Lower panels show additional views and a zoomed-in inset of the clash region.

**Supplementary Figure 7. Mutation of the PI5P4Kγ VMLLPDD motif disrupts proximal association with SNX17. A.**Representative immunoblot showing expression of HA-PI5P4Kγ wild-type and HA-PI5P4Kγ mutant (VMLLPDD→EIFLPNN) in HeLa cells. Tubulin was used as a loading control. **B.** PLA showing the proximal association between HA-PI5P4Kγ and SNX17 in HeLa cells overexpressing HA-PI5P4Kγ wild type and HA-PI5P4Kγ mutant (VMLLPDD→EIFLPNN). Representative confocal images from one of three independent biological replicates showing PLA red puncta and DAPI nuclear staining (with zoomed insets), as well as single-antibody controls are shown. Scale bar, 50 μm; inset, 20 μm.

**Supplementary Figure 8. Validation of SNX17 knockdown and integrin β1 expression in *PIP4K2C* knockout cells. A.** Representative immunoblot showing SNX17 protein levels 48 h after transfection with non-targeting siRNA (NT) or siSNX17 in control (Scr) and *PIP4K2C* knockout HeLa cells. Tubulin was used as a loading control. **B.** Representative immunoblot showing total integrin β1 protein levels in control (Scr) and *PIP4K2C* knockout HeLa cells with quantification shown. Integrin β1 signal was normalized to actin (loading control) and expressed relative to control. **C.** qPCR analysis of *ITGB1* and *PIP4K2C* mRNA expression in control and *PIP4K2C* knockout cells. Data are normalized to the housekeeping gene *GAPDH* and expressed relative to control. Bars represent mean ± SD from n = 3 independent experiments. Statistical significance was determined by one-way ANOVA followed by Tukey’s multiple comparisons test. ns, non-significant.

**Supplementary Table 1. Quantitative BioID-MS dataset for PI5P4Kα.** Proteins quantified in PI5P4Kα BioID experiments relative to BioID2 control in HeLa cells. The table reports enrichment statistics and intensity values. High-confidence hits (≥2-fold enrichment in BioID2–PI5P4Kα samples relative to myc–BioID2 control and p < 0.05) are indicated in the “Significant” column.

**Supplementary Table 2. Quantitative BioID-MS dataset for PI5P4Kβ.** Proteins quantified in PI5P4Kβ BioID experiments relative to BioID2 control in HeLa cells. The table reports enrichment statistics and intensity values. High-confidence hits (≥2-fold enrichment in BioID2–PI5P4Kβ samples relative to myc–BioID2 control and p < 0.05) are indicated in the “Significant” column.

**Supplementary Table 3. Quantitative BioID-MS dataset for PI5P4Kγ.** Proteins quantified in PI5P4Kγ BioID experiments relative to BioID2 control in HeLa cells. The table reports enrichment statistics and intensity values. High-confidence hits (≥2-fold enrichment in BioID2–PI5P4Kγ samples relative to myc–BioID2 control and p < 0.05) are indicated in the “Significant” column.

**Supplementary Table 4. Exact p-values for all statistical comparisons.** Summary of exact p-values for all statistical analyses performed in this study.
