# Supplementary figures and images for: A proximity-labeling map of PI5P4K phosphoinositide kinases interaction networks
Source: J Biol Chem. 2026 Apr 25;302(6):113071. doi: 10.1016/j.jbc.2026.113071 (PMC13218151; doi:10.1016/j.jbc.2026.113071)

A.

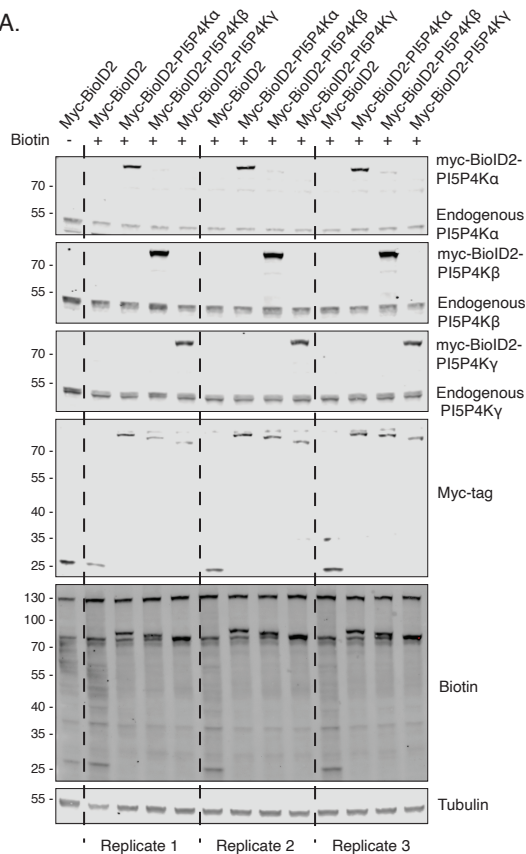

B.

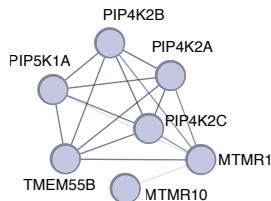

C.

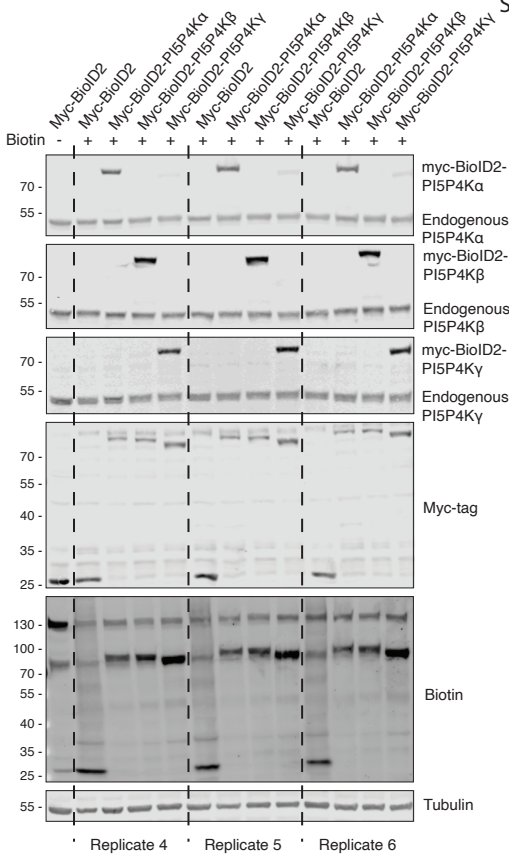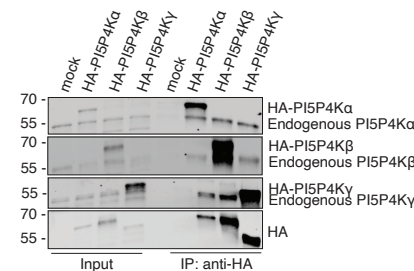

Supplement: Supplementary Figure 1 [file mmc2.pdf]

A.

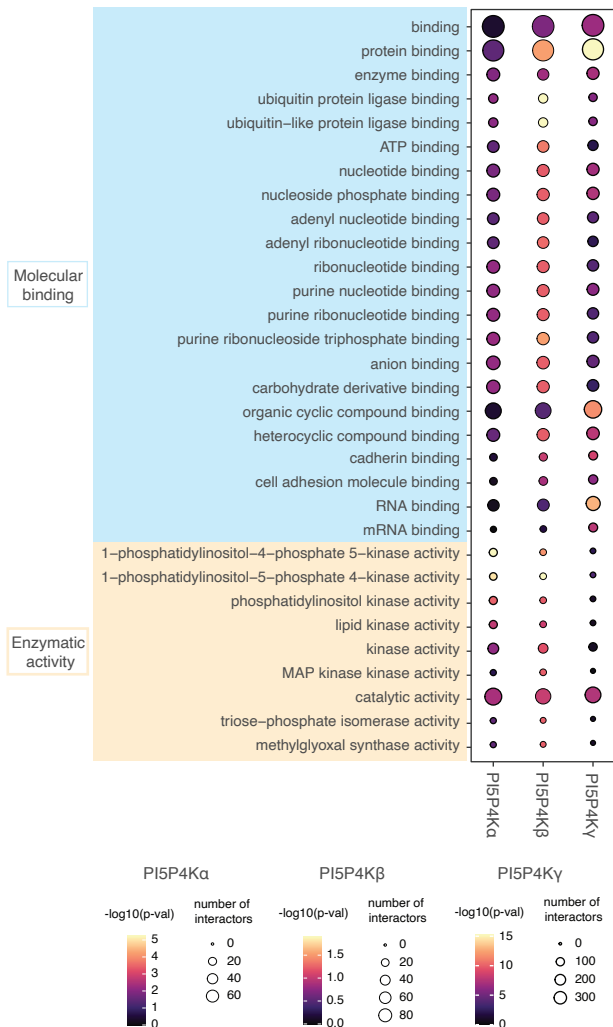

B.

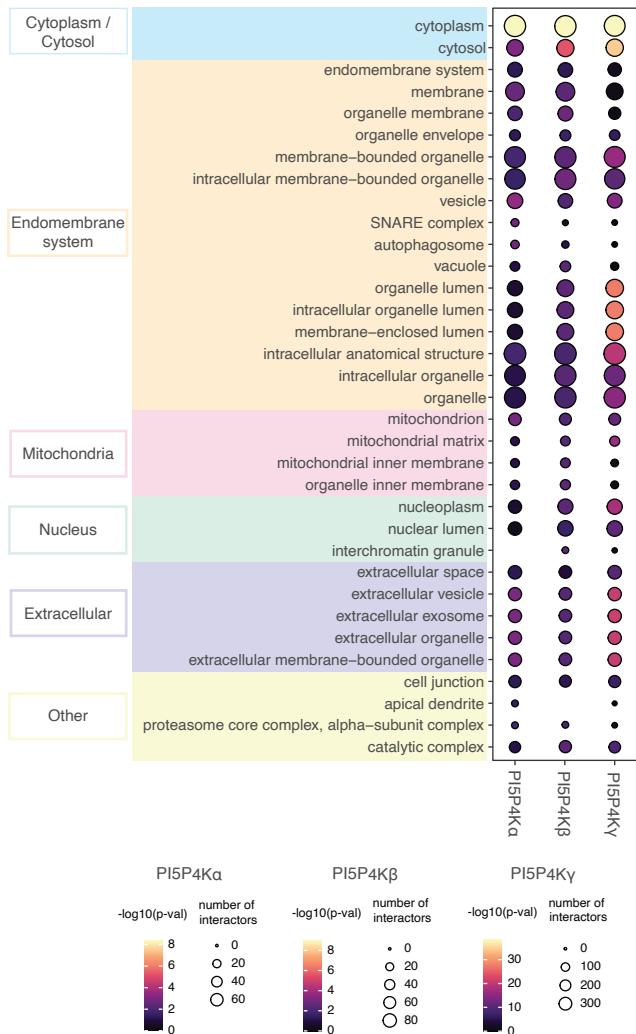

Supplement: Supplementary Figure 2 [file mmc3.pdf]

A.

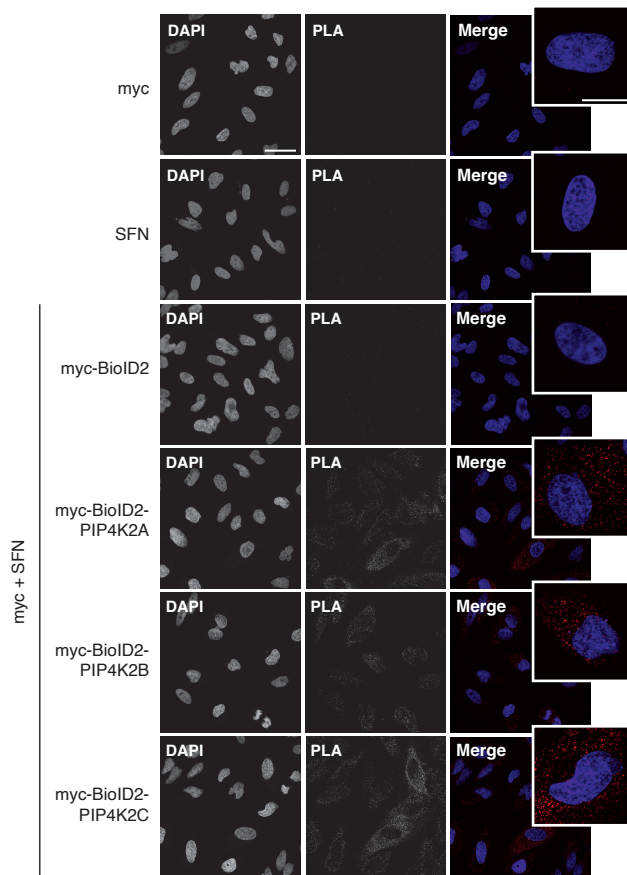

B.

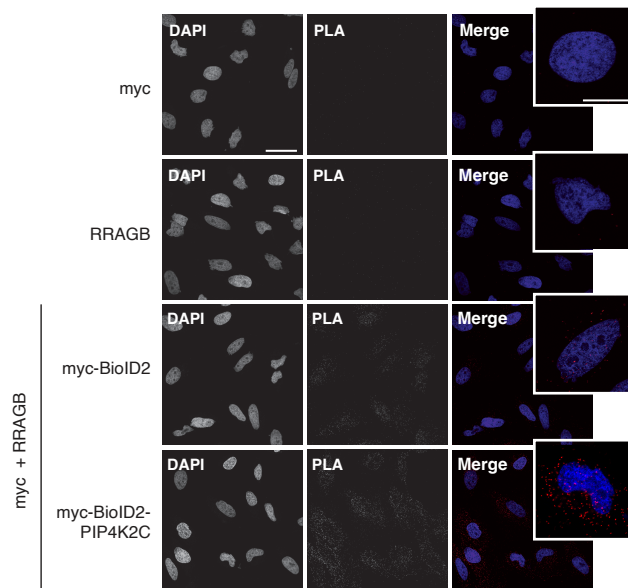

Supplement: Supplementary Figure 3 [file mmc4.pdf]

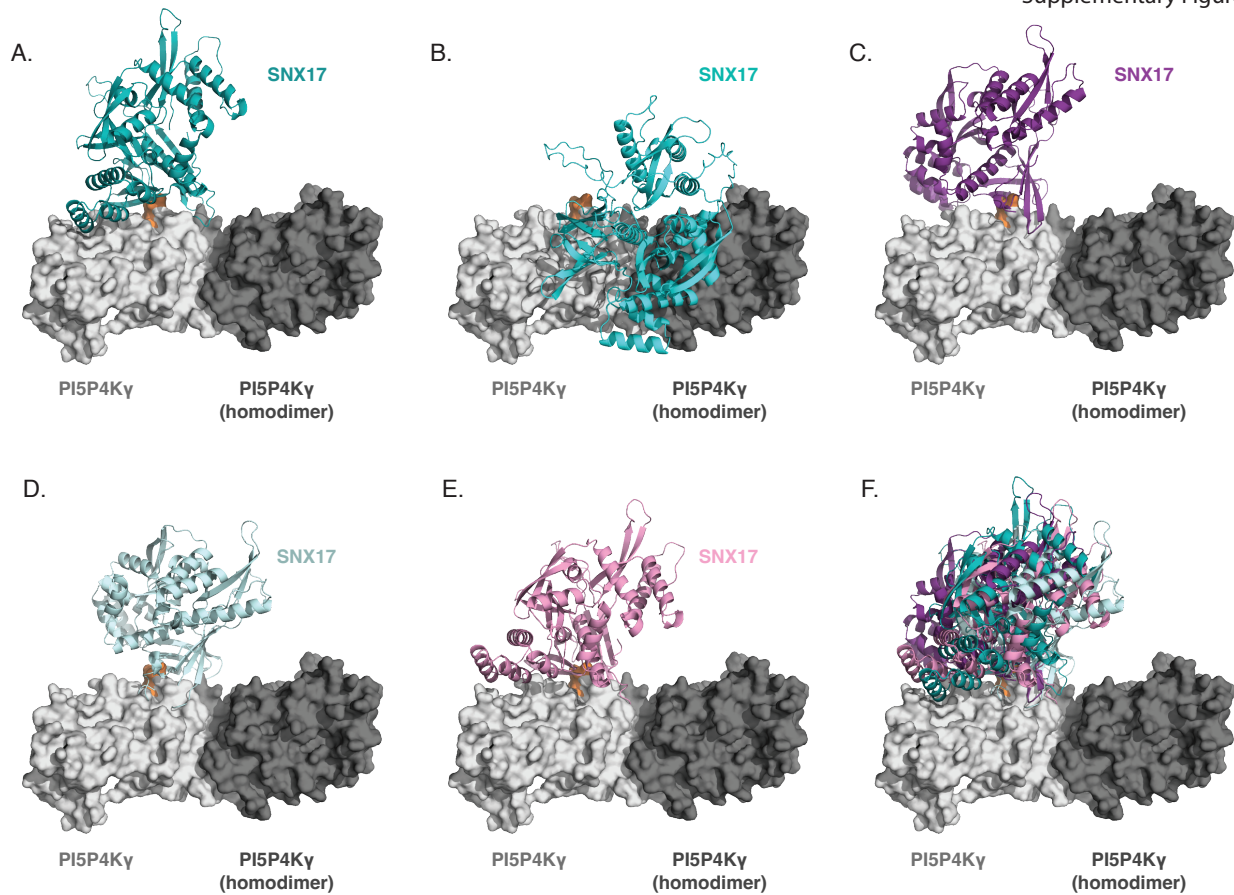

Supplement: Supplementary Figure 4 [file mmc5.pdf]

A.

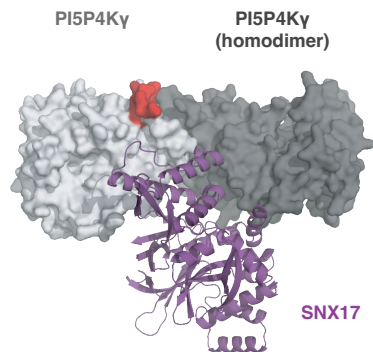

B.

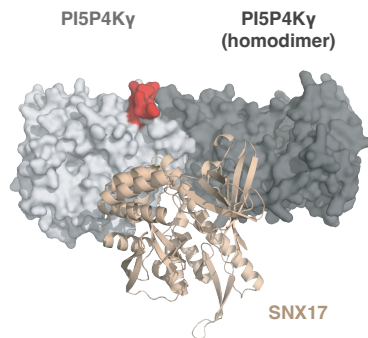

C.

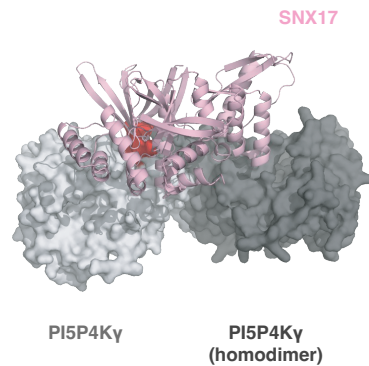

D.

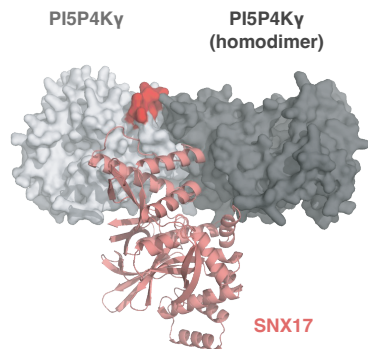

E.

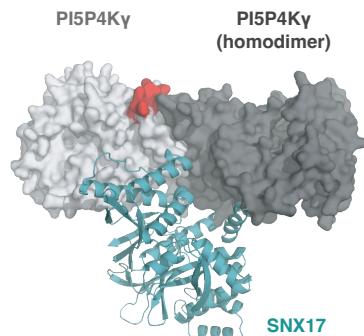

F.

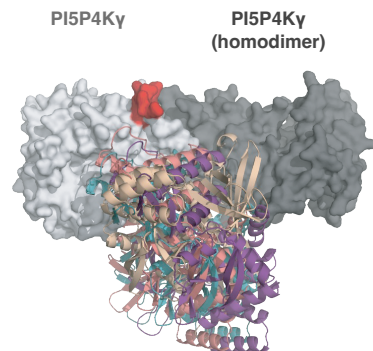

Supplement: Supplementary Figure 5 [file mmc6.pdf]

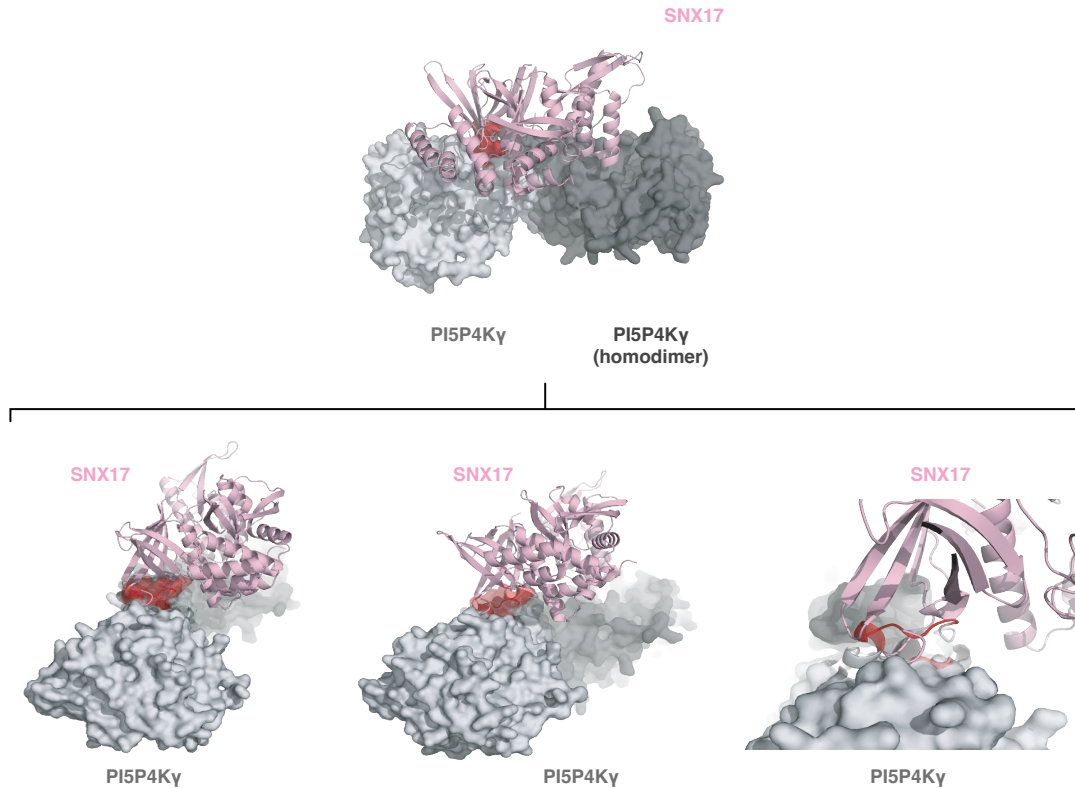

Supplement: Supplementary Figure 6 [file mmc7.pdf]

A.

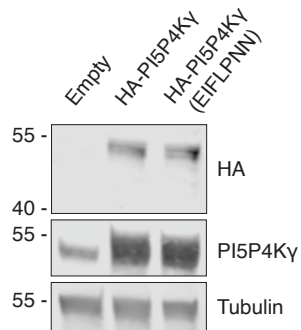

B.

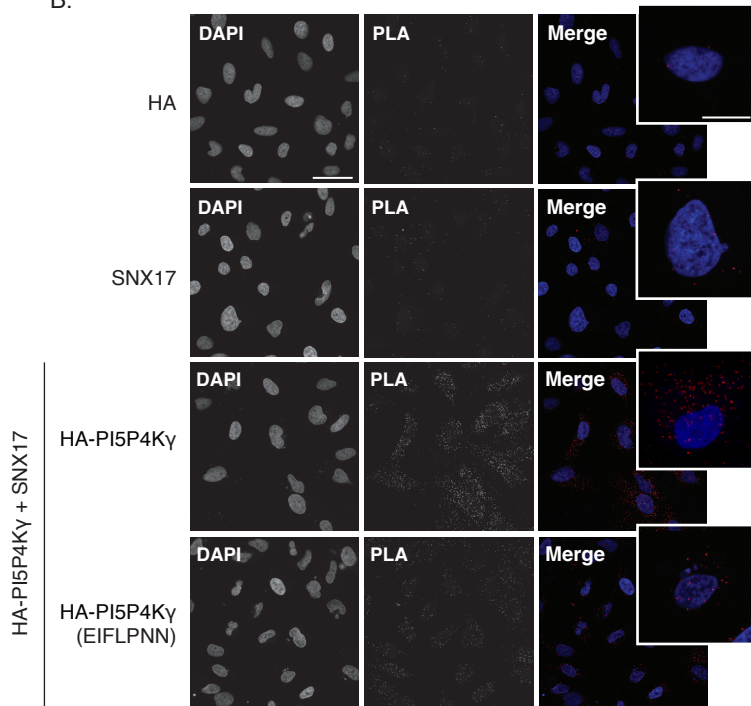

Supplement: Supplementary Figure 7 [file mmc8.pdf]

A.

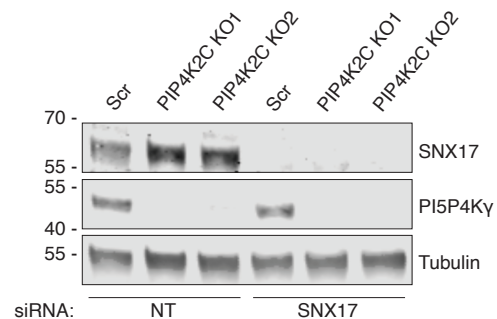

B.

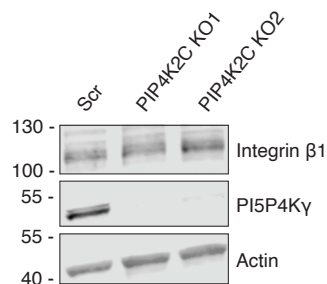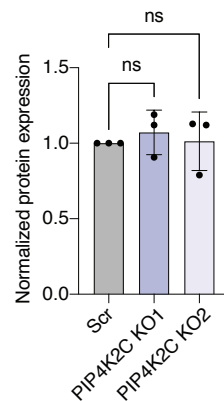

C.

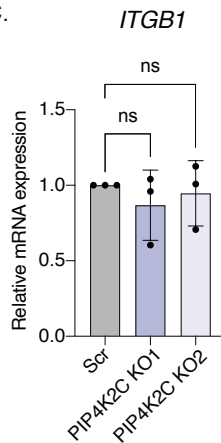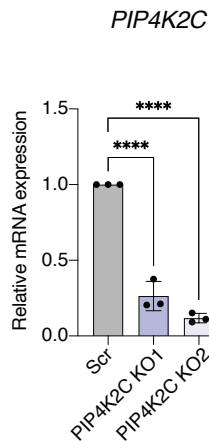

Supplement: Supplementary Figure 8 [file mmc9.pdf]
